# Successful treatment of suprasellar tumors associated with poor brain blood perfusion by severe intracranial arterial stenosis: two case reports

**DOI:** 10.1186/1756-0500-6-499

**Published:** 2013-12-01

**Authors:** Yoshikazu Ogawa, Teiji Tominaga

**Affiliations:** 1 Department of Neurosurgery, Kohnan Hospital, 4-20-1, Nagamachiminami, Taihaku-ku, Sendai, Miyagi 982-8523, Japan; 2 Department of Neurosurgery, Tohoku University Graduate School of Medicine, Sendai, Miyagi, Japan

**Keywords:** Asymptomatic, Extended transsphenoidal approach, Severe internal carotid artery stenosis, Skull base tumor, Vascular reconstruction

## Abstract

**Background:**

Treatment strategy to prevent perioperative cerebral infarction in patients with asymptomatic severe stenosis of the internal carotid artery is not fully established.

**Case presentation:**

Two patients were treated for skull base tumor in the presence of severe stenosis of the internal carotid artery, unilateral in one patient and bilateral in the other patient. Both patients were asymptomatic but had reduced vascular reserve capacity. The extended transsphenoidal approach was planned avoiding the low perfusion pressure region, with only conventional methods of maintaining blood pressure and PaCO_2_ rather than performing prophylactic vascular reconstruction surgery, and successful tumor removals were achieved without causing further neurological or radiological deficits.

**Conclusion:**

If the surgical route is planned to avoid the distribution of stenotic vessels and low perfusion pressure, prophylactic vascular reconstruction surgery would be unnecessary. Although more experiences based on sub-classified etiology for internal carotid artery stenosis are required, various types of operations including intracranial-extracranial vascular surgery might be justified based on this principle.

## Background

No protocol has been established to prevent perioperative cerebral infarction in patients with asymptomatic internal carotid artery (ICA) severe stenosis. Antiplatelet agents are sometimes administered in addition to management of any atherosclerotic disease including hypertension, diabetes mellitus, and hyperlipidemia [1-3]. However, these procedures are not supported by high-grade evidence, and the indications for prophylactic vascular reconstruction surgery depend on individual clinical characteristics [4]. Prophylactic carotid endarterectomy and/or extracranial-intracranial bypass may reduce the perioperative risks of cerebral infarction for patients during coronary artery bypass grafting [5,6], or at least show no significant difference between the vascular reconstruction group and non-reconstruction group [7]. Similar problems present in brain tumor surgery. Most previous cases involved suboccipital fossa and cranial convexity surgery [8,9], and some cases of partially removed pituitary adenomas and craniopharyngiomas through the transsphenoidal approach [10,11]. One patient with sphenoidal ridge meningioma with bilateral ICA stenoses suffered vast cerebral infarction and severely deteriorated after surgery [12]. Undoubtedly extremely delicate surgical procedures are required, but identification of the optimum indications for prophylactic vascular reconstruction surgery will greatly contribute to post-surgical improvement in this critical situation.

We report two surgical cases of skull base tumor with severe ICA stenosis. Both patients were asymptomatic but with reduced vascular reserve capacity, and successful tumor removals were performed without neurological or radiological deficits.

## Case presentation

Case 1: A 46-year-old female was introduced to our department with severe visual disturbance. She had only light perception in the right eye and temporal hemianopsia in the left eye. She had a history of hypertension since age 30 years, and medication for diabetes mellitus and epilepsy, which had been diagnosed 5 years previously at screening for moyamoya disease. She had since been treated with an antiplatelet agent and no ischemic symptom had occurred.

**Figure 1:**
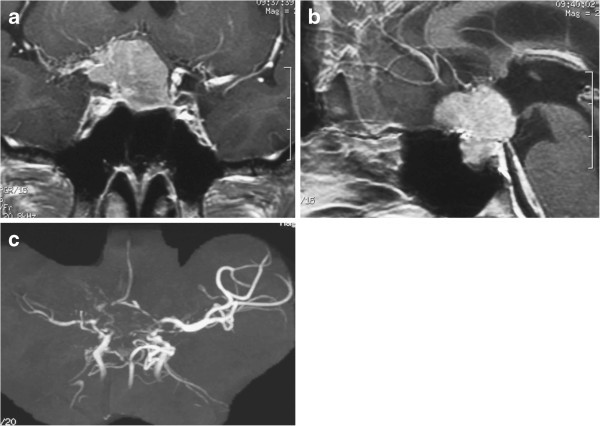
**Case 1.** Coronal **(a)** and sagittal **(b)** head MR images with contrast medium showing a well-demarcated skull base tumor, and abnormal mesh-like enhancement around the right ICA terminal and at the distal portion of the ACA. MR angiogram showing severe stenoses of the bilateral ICA terminals and moyamoya vessels **(c)**.

**Figure 2:**
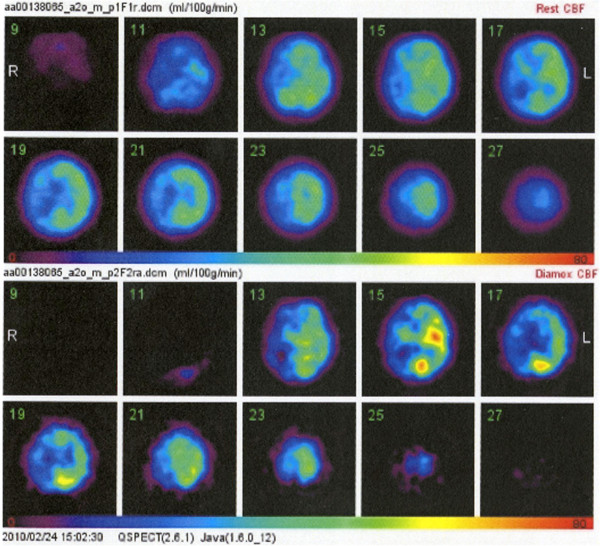
Iodine-123 N-isopropyl-p-iodoamphetamine single photon emission computed tomography scans revealing severe reduction of cerebral blood flow especially in the right hemisphere, and the steal phenomenon bilaterally after injection of acetazolamide.

Magnetic resonance (MR) imaging showed a well-demarcated skull base tumor extending from the planum sphenoidale to the diaphragm sellae. Abnormal mesh-like enhancement was seen in the right half of the tumor around the right ICA terminal, and also at the distal portion of the anterior cerebral artery (ACA) in the convexity [Figure 1a, b]. MR angiography showed severe stenoses in the bilateral ICA terminals and moyamoya vessels around the horizontal segment of the ACA and the middle cerebral artery (MCA) [Figure 1c]. Iodine-123 N-isopropyl-p-iodoamphetamine single-photon emission computed tomography (SPECT) using the autoradiographic method indicated reduced cerebral blood flow (right ACA distribution/right MCA distribution 38.4/31.4 ml/100 g/min, left ACA distribution/left MCA distribution 39.7/43.1 ml/100 g/min), and acetazolamide administration disclosed the steal phenomenon (right ACA distribution/right MCA distribution 33.9/28.9 ml/100 g/min, left ACA distribution/left MCA distribution 39.1/42.5 ml/100 g/min) [Figure 2].

**Figure 3:**
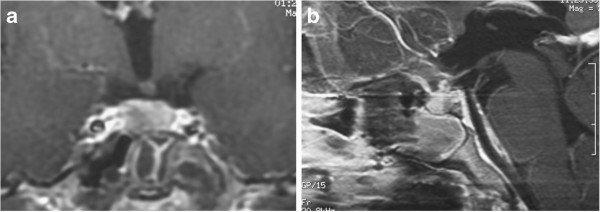
**Case 1.** Postoperative coronal **(a)** and sagittal **(b)** MR images revealing gross total removal of the tumor.

Tuberculum sellae meningioma was suspected and surgery was planned through the extended transsphenoidal route, which could avoid the low perfusion pressure region. Somatosensory evoked potentials were monitored by left tibial and median nerve stimulation throughout the surgery. PaCO_2_ was maintained over 40 mmHg, and hematocrit of peripheral blood over 35% (37.4% of preoperative state). Mean arterial pressure was maintained within the ranges between 75 and 95 mmHg. Minute feeding arteries around the tumor were selectively coagulated and devascularized one by one, and Simpson’s grade 2 removal was achieved. The patient recovered consciousness soon after the operation, and postoperative head MR imaging showed no ischemic lesions or evident tumor bulk [Figure 3]. She was discharged on the 12th postoperative day with no neurological or endocrinological deficits except for the same degree of visual disturbance in her right eye as detected preoperatively.

Case 2: A 19-year-old female was introduced to our hospital suffering from tumor re-enlargement. She had a history of initial treatment for craniopharyngioma, which had been detected at screening examination for low stature and moderate memory disturbance. Surgery through the transcranial basal interhemispheric approach resulted in subtotal tumor removal, and 50 Gy of fractionated irradiation to the remnant was given postoperatively. Four years later she suffered cerebral infarction in her right frontal lobe. MR angiography showed severe stenosis of the right ICA, and antiplatelet agent administration was started. No ischemic symptoms had occurred since then. Seven years after the initial treatment the tumor had re-grown, and she was introduced to our hospital.

**Figure 4:**
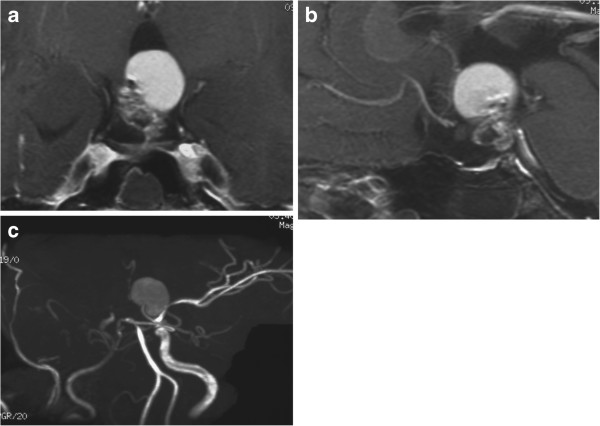
**Case 2.** Coronal **(a)** and sagittal **(b)** head MR images with contrast medium showing a dumbbell-shaped, partially solid and partially cystic tumor extending from the suprasellar cistern to the third ventricle. MR angiogram showing showed severe stenosis of the right ICA in the cavernous portion, and both A1 and M1 were only faintly visualized **(c)**.

**Figure 5:**
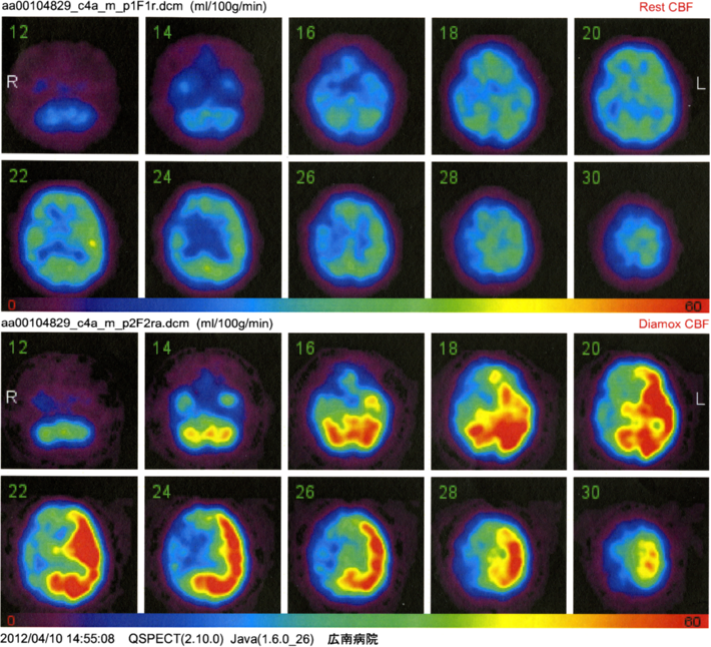
Iodine-123 N-isopropyl-p-iodoamphetamine single photon emission computed tomography scans revealing slight reduction of cerebral blood flow in the right hemisphere, and the vascular reserve capacity was markedly reduced after injection of acetazolamide.

On admission, MR imaging showed a partially ossified solid tumor in the suprasellar cistern, which had compressed the optic chiasm upwards, and a large cyst with thin wall in the third ventricle [Figure 4a, b]. MR angiography showed the right ICA was severely narrowed in the cavernous portion, and both A1 and M1 were only faintly visualized [Figure 4c]. Iodine-123 N-isopropyl-p-iodoamphetamine SPECT indicated slight reduction of cerebral blood flow (right ACA distribution/right MCA distribution 27.9/24.8 ml/100 g/min, left ACA distribution/left MCA distribution 27.9/28.4 ml/100 g/min), but acetazolamide administration indicated compromised cerebrovascular reactivity (right ACA distribution/right MCA distribution 36.1/28.3 ml/100 g/min, left ACA distribution/left MCA distribution 43.4/54.6 ml/100 g/min) [Figure 5].

**Figure 6:**
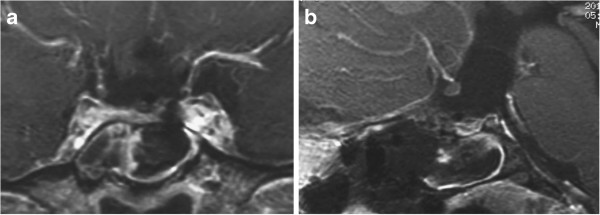
**Case 2.** Postoperative coronal **(a)** and sagittal **(b)** MR images revealing gross total removal of the tumor.

Surgery was planned through the extended transsphenoidal route to avoid the low perfusion pressure region. Somatosensory evoked potentials were monitored using left tibial and median nerve stimulation throughout the surgery. PaCO_2_ was maintained over 40 mmHg, and hematocrit of peripheral blood over 35% (44.6% of preoperative state), and mean arterial pressure was maintained within the ranges between 74 and 100 mmHg. The atrophic pituitary stalk was cut just at the transitional region to the posterior lobe, and the solid and cystic portions were removed simultaneously en-bloc. The patient recovered consciousness soon after the operation, and postoperative head MR imaging showed no ischemic lesions or evident tumor bulk [Figure 6]. She was discharged on the 12th postoperative day with no neurological deficits except for moderate recent memory disturbance, which was detected preoperatively.

## Discussion

Although a few surgical cases of brain tumor with severe ICA stenosis have been reported involving brain stem glioma and other cerebellar hemispheric tumors [8,9], partially removed pituitary adenomas and craniopharyngiomas through the transsphenoidal approach [10,11], surgery for complex and maximal tumor removal in the basal subarachnoid spaces is only rarely reported [12]. The difficulty of such procedures is mainly due to the tiny compensatory collateral circulation through reverse flow of the posterior communicating artery and/or posterior pericallosal artery, and leptomeningeal anastomosis makes the situation more complicated. Incision and detachment of the dura mater may damage this collateral circulation, and slight compression or dislocation of cerebral cortex could easily lead to extensive cerebral infarction.

Prophylactic carotid endarterectomy for the patients with severe stenosis of the ICA or extracranial-intracranial bypass for occlusion of the ICA may reduce the risk of cerebral infarction during coronary artery bypass grafting [5,6], or at least not carry a higher risk of stroke and mortality [7]. This recommendation is still controversial, but the results might vary according to the timing of the endpoint [7]. The mechanism of ischemic incidence during vascular surgery involves multiple factors including embolism, whereas the problem during tumor removal can be specifically described as maintaining local perfusion pressure. In our cases strict but simple attention to the maintenance of PaCO2 level and hematocrit adjustment brings successful removals without any ischemic events. Therefore, if the surgical route is planned to avoid the distribution depending on stenotic vessels and low perfusion pressure, prophylactic vascular reconstruction surgery would be unnecessary even in patients with low vascular reserve capacity. Selection of the surgical procedure for ruptured basilar artery aneurysm with moyamoya disease should emphasize preservation of the normal circulation [13]. Therefore, intracranial brain tumor surgery could possibly be performed safely by avoiding the low perfusion pressure distribution. Although more experiences based on sub-classified etiology for internal carotid artery stenosis are required, various types of procedures including intracranial-extracranial vascular surgery might be justified based on this principle.

## Conclusion

We successfully treated two cases of skull base tumor associated with severe stenosis of the ICA. Both patients were asymptomatic but had reduced vascular reserve capacity. If the surgical route can be planned to avoid the distribution of stenotic vessels and low perfusion pressure, prophylactic vascular reconstruction surgery would be unnecessary.

## Consent

Written informed consent was obtained from both patients for publication of this Case Report and any accompanying images. Copies of the written consents are available for review by the Editor-in-Chief of this journal.

## Competing interest

The authors declare that they have no competing interest.

## Authors’ contributions

YO performed tumor removal and analyzed the patient data, and was a major contributor in writing the manuscript. TT gave an essential suggestion and supervised this manuscript. Both authors read and approved the final manuscript.
